# Probabilistic functionality assessment of road networks for medical emergency vehicles during flooding

**DOI:** 10.1007/s11069-026-08005-z

**Published:** 2026-02-26

**Authors:** Ke He, Neil Carhart, Maria Pregnolato, Jeffrey Neal, Raffaele De Risi

**Affiliations:** 1 Department of Civil Engineering, University of Bristol, Bristol, BS8 1TR UK; 2 Department of Civil Engineering, Delft University of Technology, Delft, 2628 CD Netherlands; 3 School of Geographical Sciences, University of Bristol, Bristol, BS8 1SS UK

**Keywords:** Flood risk, Network performance, Probabilistic analysis, OpenStreetMap

## Abstract

Flood damage to road networks primarily manifests as a loss of transportation functionality. Current analyses of road network functionality loss during floods are based on specific flood scenarios. This study analyses flood risk to road networks by assessing the probability of stability loss for various vehicle types (SUVs/emergency vehicles, and cars). Eventually, a flood risk map of the road network is generated. The flood risk of each road is computed as reduced accessibility, measured in this paper via isochrones. Bristol (UK) is used as the case study area, with all hospitals as starting points to study the coverage area of emergency vehicles within a given time frame. The results indicate that road network functionality for SUVs/emergency vehicles has a lower flood risk than that for cars. Additionally, the city centre of Bristol exhibits a higher flood risk, hindering emergency medical vehicles from reaching high-risk flood areas. The findings of this research offer strategies to mitigate the impact of floods on road networks and prepare emergency medical services before flood disasters occur.

**Supplementary Information:**

The online version contains supplementary material available at 10.1007/s11069-026-08005-z.

## Introduction

Road networks connect the population to essential civil emergency services. However, the road network can be susceptible to natural hazards, especially flooding (Papilloud and Keiler 2021). Flooding can affect functionality, reducing traffic efficiency (e.g., reducing travel speed or accessibility (Rebally et al. 2021). Furthermore, the effects of flooding on the functionality of individual roads can propagate throughout the entire road network, resulting in cascading impacts and causing traffic congestion (Dong et al. 2022a, b; Kasmalkar et al. 2020; Pyatkova et al. 2019), leading to increased travel times for both regular and emergency vehicles. For instance, the area accessible to ambulances departing a hospital may shrink significantly. It is, therefore, clear that flooding affects people’s access to essential civil amenities.

Research on modelling flooding events has reached a high level of maturity. For example, the Hydrologic Engineering Centre’s River Analysis System (HEC-RAS), developed by the U.S. Army Corps of Engineers, is widely used in scientific research to model river hydraulics and floodplain dynamics (U.S. Army Corps of Engineers 2019). The two-dimensional flood modelling model (e.g. FLO-2D) is applicable to diverse flood-related research, including urban flooding and riverine inundation scenarios (Li et al. 2021; Liu et al. 2022). The hydrodynamic model LISFLOOD-FP, which is based on raster grid representation and capable of simulating inundation caused by flood events (Bates and De Roo 2000), has been extensively utilised in studies focusing on urban drainage modelling (Wu et al. 2018; Yang et al. 2022), coastal flooding (Hirai and Yasuda 2018; Seenath 2018) and network impact (Pregnolato et al. 2022).

Furthermore, research investigating the relationship between flood hazards and road network functionality is becoming increasingly mature. The standard approach to assessing the functionality loss of the road networks consists of integrating the flood hazard maps and road network features together (De Risi et al. 2015; Risi et al. 2018a, 2020; He et al. 2023; Jalayer et al. 2014; Kalantari et al. 2017; Pregnolato et al. 2017; Zhang and Alipour 2019). Some studies have explored the deterministic resistance of vehicles to flooding under varying flood intensities. Several relationships among vehicle stability, flood depth, and flow velocity are reported in the literature (Shu et al. 2011; Teo et al. 2012; Toda et al. 2013; Xia et al. 2011, 2013, 2016). These studies concluded that flooding impacting the vehicle laterally (rather than frontally) has the most critical impact on vehicle stability (Wang et al. 2021). According to He et al. (2023), overall network performance is assessed by integrating flood hazards, road network topology, and vehicle vulnerability to evaluate the impact severity of exposed links and the functionality reduction of the entire network. Alabbad et al. (2021) examined variations in the reachable/accessible area from residential properties to critical amenities at the 100-year and 500-year flood return periods to identify the most vulnerable roads under different flood hazards. To evaluate the possibility of different vehicles on inundated roads, roadworthiness, based on vehicle design and stability in flood, was used as an indicator of road performance (He et al. 2024).

The studies discussed above are predominantly deterministic and scenario-based, typically anchored to specific return periods and neglecting key epistemic and aleatory uncertainties. Unlike depth–damage models used to relate flood hazard to building losses (e.g., Chang et al. 2009; Dias et al. 2018; Karambiri et al. 2018; Galasso et al. 2021), existing functional relationships for vehicle stability under flooding do not account for uncertainties. A few studies express vulnerability probabilistically through fragility curves, i.e., the conditional probability of exceeding or reaching a given damage state for a specified intensity measure (De Risi et al. 2013). In principle, such fragility functions should be hazard-agnostic and therefore independent of flood return period (Beevers et al. 2022). However, truly hazard-agnostic fragility models for vehicle instability in flood conditions have yet to be developed. Beyond these limitations, the literature lacks any risk metric capable of assessing road-network functionality using flood hazard maps across multiple return periods in combination with probabilistic vulnerability models. Finally, it is worth noting that, in the context of building flood risk, approaches that couple 1D/2D hazard models with microscale damage models have proven most effective (Apel et al. 2008).

This study addresses several of the gaps identified above. First, we introduce a procedure for computing flood hazard curves that integrates hazard information across all available return periods. We then present an innovative method for developing functionality fragility curves for individual road links. Building on these components, a fully probabilistic framework is proposed to convolve hazard and vulnerability and produce risk maps for the entire road network. These maps are subsequently used to derive isochrones for medical emergency vehicles serving major hospitals. The methodology is demonstrated through a case study of the city of Bristol, UK. Finally, the article discusses the benefits of a probabilistic approach compared with traditional deterministic and scenario-based analyses, extending insights from the existing literature (Evans et al. 2024; He et al. 2023; Wang et al. 2021).

This paper comprises five sections. Following the introduction, Sect. 2 describes the research methodology, including the flood hazard curve, the derivation of fragility curves, risk convolution, and functionality loss assessment. Section 3 shows the procedure results for Bristol; isochrone areas associated with functionality loss for medical emergency service vehicles are also presented. Section 4 discusses the advantages of the probabilistic approach and elucidates the significance of future flood risk management. Eventually, Sect. 5 wraps up the main takeaway messages, emphasising the limitations.

## Methodology

**Fig. 1:**
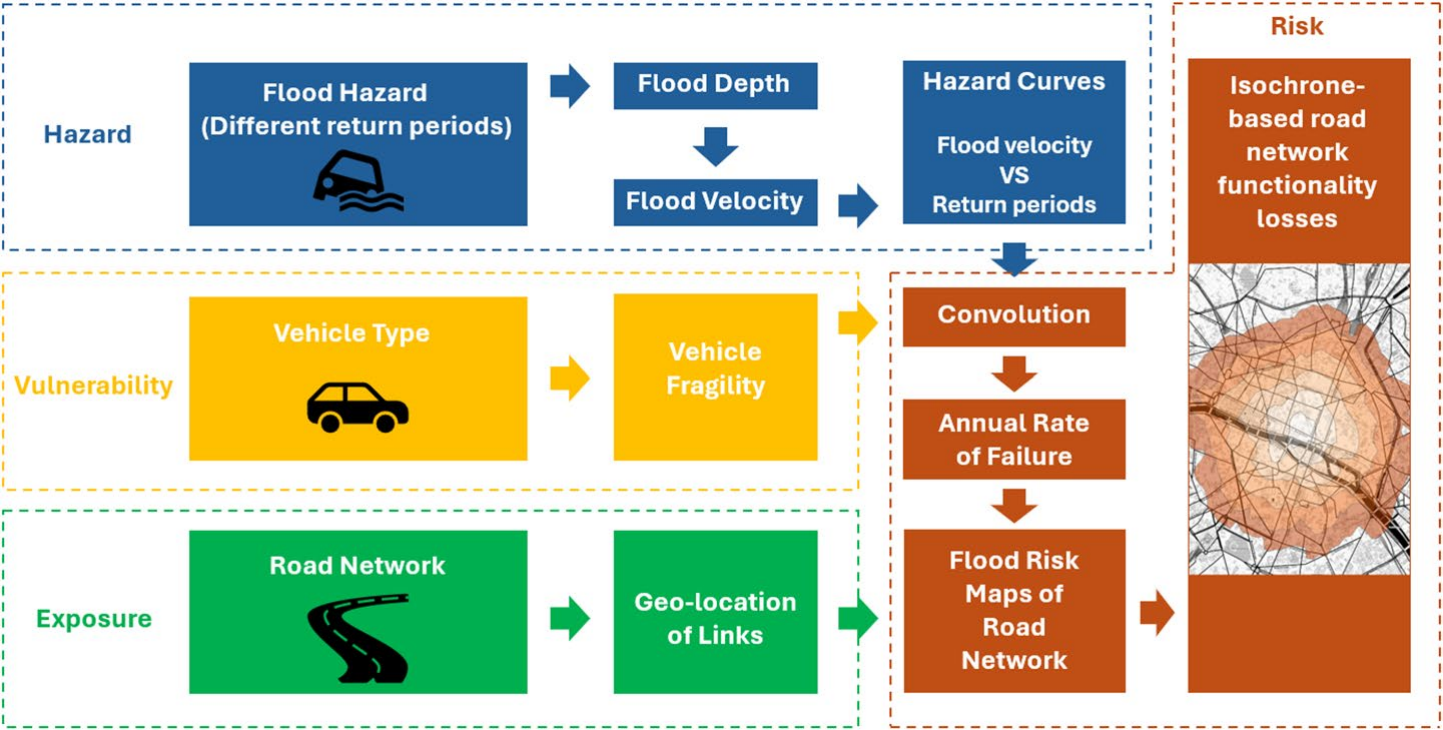
Work flowchart for road network risk map and functionality assessment

This research methodology is divided into three main parts: (i) flood hazard curve derivation, (ii) analysis of the fragility of road networks of different vehicles based on flood flow velocity, and (iii) convolution of flood hazard and road network fragility to assess the risk and functionality losses of road networks. It is worth mentioning that this paper described flooding thoroughly using both flood depth, $\:{h}_{f}$, and flood velocity, $\:{v}_{f}$. These two quantities will be referred to as Intensity Measures (IMs) in the following sections. Figure 1 illustrates the workflow of this study. Detailed explanations for each part will follow.

### Flood risk definition

De Risi et al. (2013) proposed a general probabilistic method for flood risk assessment, as shown in Eq. 1. $\:{\lambda\:}_{LS}$ denotes the annual exceedance rate of a given limit state (*LS*). In this study, *LS* refers to the functional loss across different vehicle types. $\:P\left(LS\right|{v}_{f})$ is the vehicle fragility function concerning the *LS*, i.e., the conditional probability of reaching or exceeding a specific LS conditioned on a specific value of flood intensity measure (in this case, the flood velocity $\:{v}_{f}$). $\:\left|d\lambda\:\right({v}_{f}\left)\right|$ is the flood hazard term derived as the absolute value of the hazard increment for all flood velocities $\:{v}_{f}$. Eventually, the flood risk of each link or node in a transportation network contributes to the network’s overall flood risk.

1 $$\:{\lambda\:}_{LS}=\underset{{v}_{f}}{\overset{}{\int\:}}P\left(LS\right|{v}_{f})\cdot\:|d\lambda\:\left({v}_{f}\right)|$$

### Flood hazard curve

The hazard curve represents the mean annual rate of observing a specific hazard intensity measure in a certain observation time in a particular area (Kinyua 2018). The primary factors contributing to flood damage are flood depth and flow velocity; indeed, these are among the most commonly used measures of flooding intensity in the literature (Kreibich et al. 2009). Unlike buildings and bridges, the impact of a flood on road networks lies in functional disruption rather than structural integrity, specifically affecting the stability of vehicle movement. The drag force generated by water flow can affect vehicle stability (Martínez-Gomariz, 2017; Al-Qadami et al. 2021). Therefore, following the findings of He et al. (2023), this study derives a flood hazard curve based on flood flow velocity $\:{v}_{f}$.

Flood data is typically available in raster format. Sometimes, only flood depth is available. In such a case, the flood depth can be converted to flood flow velocities using an approximation for shallow water (Ponce & Simons 1977), as shown in Eq. 2. However, for low-gradient floodplains where the flow is subcritical, this equation is likely to overestimate the actual velocity, whilst for steep catchments where the flow is supercritical, this equation will underestimate the velocity. So, ideally, flood velocity would be computed by hydrodynamic models. Equation 2 uses a simple model to estimate flood velocity as a function of flood depth.

2 $$\:{v}_{f}=\sqrt{g{h}_{f}}$$

where $\:{v}_{f}$ is the expected flood flow velocity, $\:{h}_{f}$ is the flood depth, and $\:g$ is the gravity acceleration (9.81 m/s^2^).

In this study, the flood hazard curve for each road is fitted, as per Jalayer et al. (2016), using Eq. 3 to the available flood data for different flooding scenarios (i.e., flood maps corresponding to multiple return periods).

3 $$\:\lambda\:\left({v}_{f}\right)=\:{K}_{0}\times\:{{v}_{f}}^{K}$$

where $\:{K}_{0}$ and $\:K$ are two parameters of the flood hazard curve. $\:\lambda\:\left({v}_{f}\right)$ is the annual frequency of exceeding a given flood flow velocity. In this study, the flood flow velocities corresponding to different return periods are fitted with Eq. 3 to determine the flood hazard parameters $\:{K}_{0}$ and $\:K$. These parameters are estimated using the Maximum Likelihood Estimation (MLE) method.

**Fig. 2:**
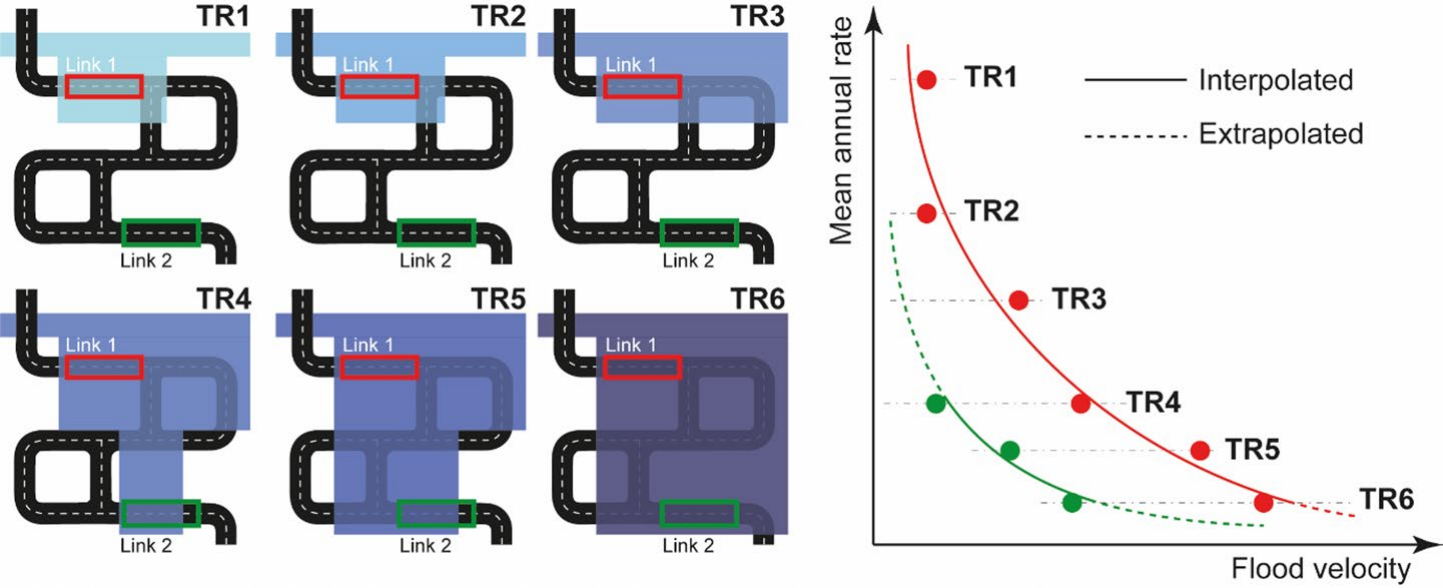
Flood hazard curve deviation example with flood data available for all return periods (Link 1, red curve). Flood hazard curve deviation example with missing flood data for some return periods (Link 2, green curve)

It is important to emphasise that, given the effect of the topography on flooding (De Risi et al. 2015) and the location of the roads, flood data is not always available for every element of the network (De Risi et al. 2020). In simpler terms, for a road network, not every road is affected by flooding for all return periods. For example, roads located in high-elevation areas and far from rivers would be affected only in extreme cases, for which there is no available data. Therefore, the flood hazard curve will be fitted only to the available data, and extrapolation will be necessary in some cases, as observed in other studies (De Risi et al. 2018b). Figure 2 shows a schematic example of the total and partial availability of flood data.

### Fragility functions for vehicle stability

A key novelty of this research is the derivation of fragility curves for vehicle stability using data from the literature. The fragility curve represents the conditional probability of reaching or exceeding a specific damage state conditioned on a prescribed intensity measure (De Risi et al. 2013). The fragility of vehicles in a flood can be expressed as $\:P\left(LS\right|{v}_{f})$ according to Eq. 4:

4 $$\:P\left(LS|{v}_{f}\right)=\phi\left(\frac{\mathrm{l}\mathrm{o}\mathrm{g}\left({v}_{f}\right)-{\mu\:}_{log}}{{\sigma\:}_{log}}\right)$$

where $\:\phi( \cdot \:)$ is the operator for the Normal cumulative distribution function (CDF), $\:{v}_{f}$ is the generic value of flood velocity and $\:{\mu\:}_{log}$ and $\:{\sigma\:}_{log}$ are the mean value and standard deviation of the logarithm of the critical flood velocities. In this study, since the fragility of vehicles is caused by the product of uncertainties of flood intensity and the inherent characteristics of the vehicles, and since the velocity is considered as always positive, the probability distribution of vehicle fragility can be represented using a lognormal distribution (Faber, 2012).

The critical flood velocity is the maximum flood velocity that a vehicle can withstand in a given flood depth, beyond which the vehicle will become unstable (Martínez-Gomariz, 2017; Wang et al. 2021; He et al. 2023). The critical flood velocity for different types of vehicles varies with vehicle characteristics and the coefficient of friction between tyres and the ground (Wang et al. 2021; Lazzarin et al. 2022). The critical flood velocity for a given vehicle also depends on the direction of the incoming flood relative to the vehicle’s travel (Qadami et al., 2021). This study considers the critical flood velocity of a vehicle for two extreme incoming flood directions: perfectly parallel to the vehicle’s direction of travel and perpendicular to the vehicle’s direction of travel (Eqs. 5 and 6). In addition, this study uses two types of vehicles to represent most vehicles in the road network: SUVs (Sports Utility Vehicles) and cars. It is important to emphasise that emergency vehicles are treated as SUVs in this paper, since they are usually larger than typical private cars. Table 1 provides the parameters for these two vehicles used to calculate the mean critical flood velocities in Eqs. 5 and 6.

5 $$\:{u}_{c,\left|\right|}={\alpha\:}_{\left|\right|}({h}_{f}/{h}_{c}{)}^{\beta\:\left|\right|}\sqrt{2g{l}_{c}({\rho\:}_{c}{h}_{c}/\left({\rho\:}_{f}{h}_{f}\right)-{R}_{f})}$$

6 $$\:{u}_{c,\perp\:}={\alpha\:}_{\perp\:}({h}_{f}/{h}_{c}{)}^{\beta\:\perp\:}\sqrt{2g{b}_{c}({\rho\:}_{c}{h}_{c}/\left({\rho\:}_{f}{h}_{f}\right)-{R}_{f})}$$

where $\:{u}_{c,\left|\right|}$ and $\:{u}_{c,\perp\:}$ are the critical flood velocities of parallel and perpendicular incoming flood, respectively. α and β are parameters relative to vehicle and road surface features. $\:{h}_{f}$ and $\:{\rho\:}_{f}$ are flood depth and density. $\:{h}_{c}$, $\:{b}_{c}$, and $\:{l}_{c}$ represent the dimensions of the vehicle – height, width, and length scaled to account for the experimental evidence. $\:{\rho\:}_{f}$ is the floodwater density. $\:g$ is the gravity acceleration. $\:{R}_{f}=\:{h}_{c}{\rho\:}_{c}/\left({h}_{k}{\rho\:}_{f}\right)$, where $\:{h}_{k}$ is the flood depth at which the vehicle starts floating (Dong et al. 2022a, b; Xia et al. 2013). It should be noted that in previous studies (Xia et al. 2013) critical vehicle velocities were not derived using full-scale vehicles, but rather from experiments with geometrically scaled die-cast vehicle models. Accordingly, all vehicle parameters provided in Table 1 were scaled proportionally to preserve vehicle density and to reproduce vehicle inundation conditions as realistically as possible.

**Table 1:** Parameters for critical flood velocity derivation for suvs and cars (Wang et al. 2021)

Vehicle		Parameters								
		α	β	$\:{h}_{c}$ (m)	$\:g$ (m/s^2^)	$\:{l}_{c}$ (m)	$\:{\rho\:}_{c}$ (kg/m^3^)	$\:{\rho\:}_{f}$ (kg/m^3^)	$\:{R}_{f}$	$\:{h}_{k}$ (m)
SUV	∥	0.438	-0.219	1.737	9.8	5.089	203	1000	0.551	0.67
car	∥	0.212	-0.562	1.48	9.8	4.945	170.44	1000	0.65	0.45
		α	β	$\:{h}_{c}$ (m)	$\:g$ (m/s^2^)	$\:{b}_{c}$ (m)	$\:{\rho\:}_{c}$ (kg/m^3^)	$\:{\rho\:}_{f}$ (kg/m^3^)	$\:{R}_{f}$	$\:{h}_{k}$ (m)
SUV	⟂	0.367	-0.451	1.737	9.8	1.983	203	1000	0.551	0.67
car	⟂	0.492	-0.344	1.48	9.8	1.845	170.44	1000	0.65	0.45

Regarding the logarithmic standard deviation of the critical flood velocity ($\:\sigma\:$), this study innovatively uses previous studies’ experimental data (Xia et al. 2013) of vehicle critical flood velocity to assess the uncertainty for deriving the standard deviation; this will be presented in more detail in Sect. 3.2. The goodness of fit between the regression curves and the experimental depth–velocity data was quantified using the coefficient of determination $\:{R}^{2}$, which represents the proportion of variance in the observations explained by the fitted model. The logarithmic standard deviation is calculated by investigating the residuals between the experimental data and the critical flood velocity function.

### Risk assessment and functionality loss

In principle, the fragility functions should be hazard-agnostic, i.e., independent of the flood return period. Unfortunately, the median of the fragility curves, as evident from Eqs. 5 and 6, depends on flood depth corresponding to a specific return period. Therefore, Eq. 4 becomes $\:P\left(LS|{v}_{f},{h}_{f}\right)$. Two approaches can be used to solve this double-dependency problem.

The first approach consists of computing the hazard curve for flood depth as per Sect. 2.2, and then extending the integration of Eq. 1 to two dimensions over all possible values of flood depth and flood velocity; this is the most rigorous approach. The second approach is more straightforward and is based on the extreme value theory, which is generally used to characterise the probabilistic occurrence of flood return periods. This approach is an approximation to the full 2D hazard–fragility convolution. Specifically, Eq. 1 can be computed for all possible scenarios using the scenario flood depth for the fragility curve. Therefore, the mean annual rate will be a function of the return period (i.e., $\:{\lambda\:}_{LS}\left[{h}_{f}\left({T}_{R}\right)\right]$ which is the conditional rate under the specific scenario $\:{T}_{R}$). To remove such an unwanted dependency, the total probability law can be used. Specifically, the overall rate $\:{\lambda\:}_{LS}\:$can be computed as:

7 $$\:\stackrel{-}{{\lambda\:}_{LS}}=\mathbb{E}\left[{\lambda\:}_{LS}|Scenario\right]=\sum\:_{i=1}^{\#{T}_{R}}{\lambda\:}_{LS}\left[{h}_{f}\left({T}_{R,i}\right)\right]\cdot\:{p}_{{T}_{R,i}}$$

where $\:\stackrel{-}{{\lambda\:}_{LS}}$ is the mean annual rate obtained as the expected value ($\:\mathbb{E}$) of the conditional rates. This can be achieved by weighting the specific conditional rates and the discretised probability masses from the extreme-value model ($\:{p}_{{T}_{R,i}}$). Eventually, the obtained mean annual rate is independent of any specific flood return period.

In this paper, 1 year is used as the observation time to calculate the annual probability of failure. This probability is the likelihood of functional loss in the road network over 1 year. In this study, the Poisson process distribution is employed to calculate the annual probability of failure. The Poisson distribution can be applied to various engineering-related problems, particularly non-overlapping and mutually independent events (Faber, 2012). The expression of the annual probability of failure is described by Eq. 8:

8 $$\:{p}_{f}=1-\mathrm{e}\mathrm{x}\mathrm{p}(-\stackrel{-}{{\lambda\:}_{LS}}*t)$$

where $\:{p}_{f}$ is the annual failure probability, while *t* is the observation time, equal to 1 year. This probability will be used as a reduction factor of the full-capacity properties of the specific affected link. The functional loss of the road network is reflected primarily in its traffic performance. A time-isochrone represents the area reachable within a specified travel time (e.g., 10 min) from a given origin under a particular transport mode (cars, bicycles, public transport) (Alexandros Efentakis et al., 2013; Akinsowon 2021; McKenzie 2022; Zeng et al. 2014). Evaluating road performance using travel-time maps is known as time-based isochrone analysis (Coles et al. 2017; Green et al. 2017; Bainbridge, 2021; Śleszyński et al. 2023). Isochrones capture network accessibility and reveal cascading effects associated with network disruption, while avoiding the need for dynamic traffic simulation. As such, they are computationally efficient and suitable for near-real-time applications. In this study, time-based isochrones for hospitals are of primary interest. For emergency vehicles (assumed to behave similarly to SUVs), reduced network functionality manifests as increased travel time and decreased accessibility.

## Results

**Fig. 3:**
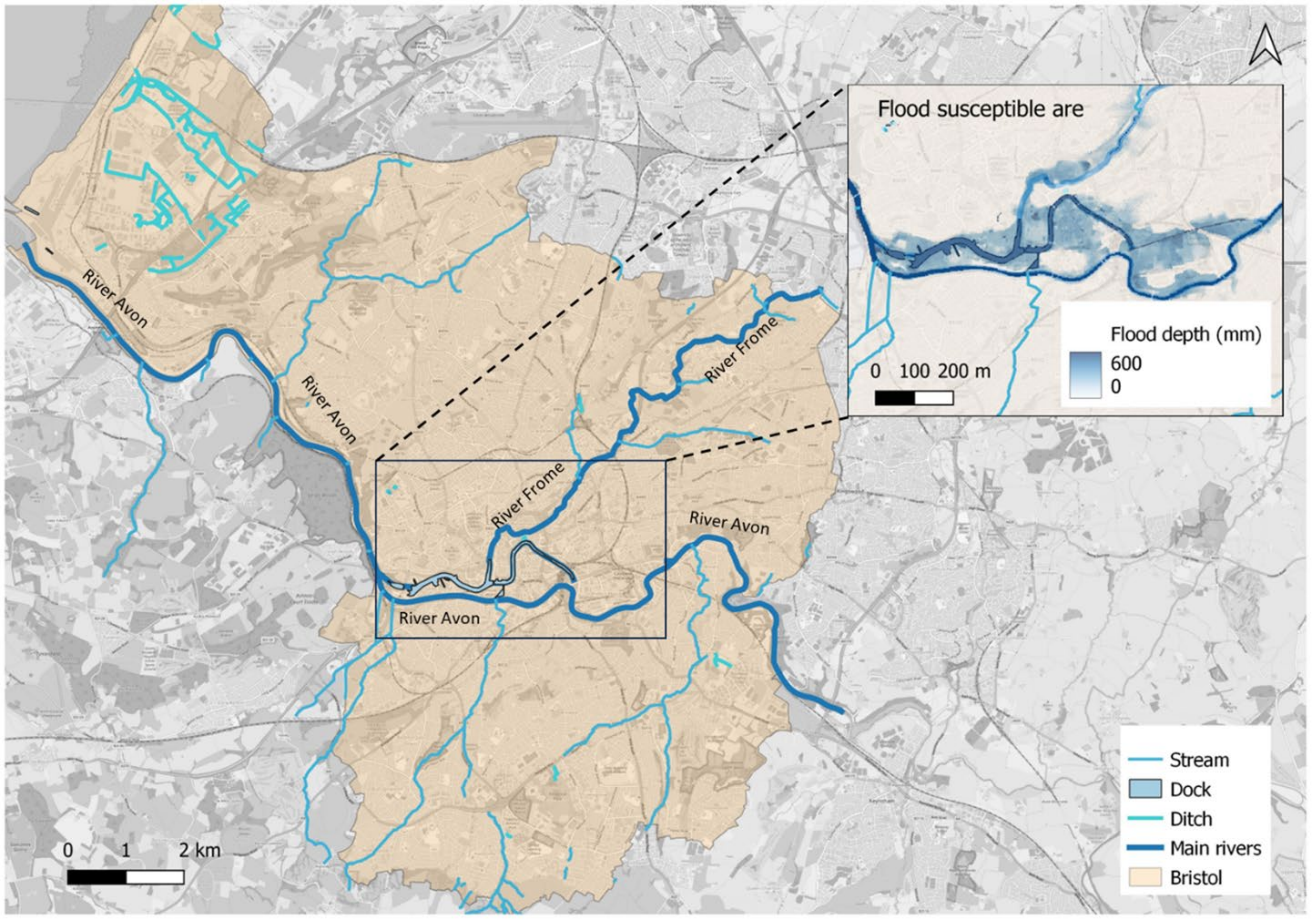
The map of Bristol, water bodies, and the flood susceptible areas

The city of Bristol, located in the West of England, is the designated case study area. Figure 3 shows the region, including its major rivers, water bodies, and areas affected by a 200-year flood scenario. The city centre is traversed by the west-east flowing river Avon, while the river Frome courses northward from the city centre. Characterised by its susceptibility to flooding, Bristol has experienced recurrent inundation within its network (Arup 2020), including pluvial flooding.

The Bristol flood data comprises two-dimensional flood-depth raster maps with a 10-meter resolution, simulated using the LISFLOOD-FP 2D hydrodynamic model (Bates et al. 2023). This study considers flood return periods of 5, 10, 20, 50, 75, 100, 200, 250, 500, and 1000 years. The flood data for each return period represent the maximum flood depth at each point on the map. The available flood data do not include flood flow velocities. Therefore, flood flow velocities are computed by using the relationship shown in Eq. 2.

The topological structure of Bristol’s road network is built using open-source OpenStreetMap data processed through QGIS. The road network primarily consists of two components: nodes and links. Nodes represent intersections and endpoints of roads within the road network, while links represent the roads and bridges. This study integrates road network topology and flood raster data to analyse the flood risk experienced by each road. It is important to note that a road may overlap multiple raster grids, and the flood depth in each grid may vary, indicating that the road faces different flood risks. To streamline calculations, the maximum flood depth along each road is selected to represent the overall flood risk for that road. This assumption is deemed reasonable, as the functionality of the entire road is compromised once any part of it is inundated to the extent that vehicular passage is impeded. This overestimation, accounting for the worst-case scenario, enhances the safety of vehicular travel on the road network.

This section discusses the flood risk maps derived using the methodology outlined in Sect. 2. In this study, all hospitals serving as emergency medical facilities are considered nodes and designated departure points for ambulances. The isochrone coverage areas of hospitals with emergency services (ambulances) reflect the degradation in the performance of Bristol’s road network following flooding.

### Flood hazard curves

**Fig. 4:**
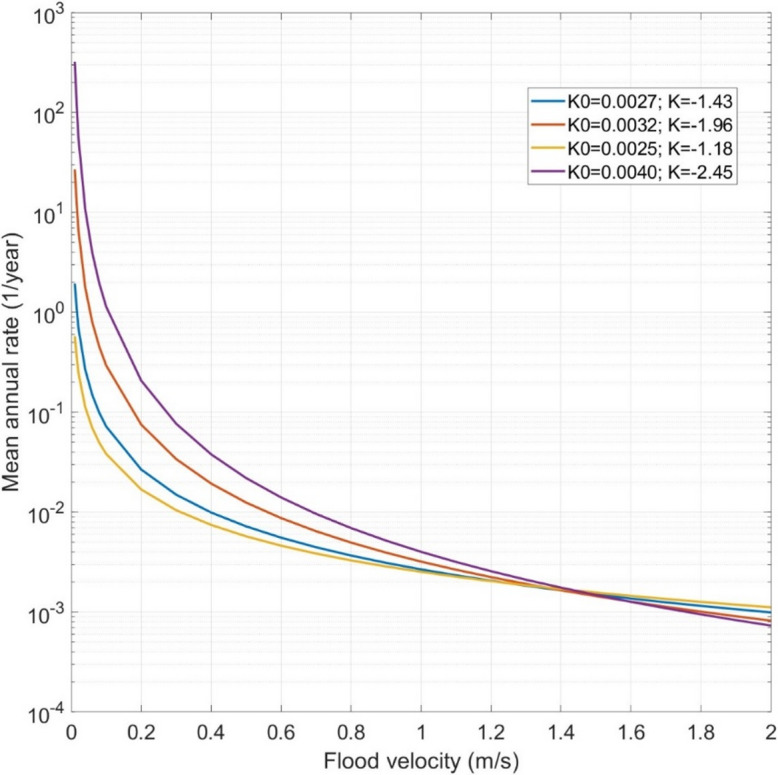
Example of the hazard curves fitted with Eq. 3

This study used the flood hazard curves for each road, calculated for 10 scenarios corresponding to 10 return periods (i.e., 5, 10, 20, 50, 75, 100, 200, 250, 500, and 1000 years). The parameters $\:{K}_{0}$ and $\:K$ are computed for each inundated road, as explained in Sect. 2.2. Examples of flood hazard curves of several roads are presented in Fig. 4. Variations among curves reflect differences in the parameter pair ($\:{K}_{0}$,$\:\:K$), which governs the shape of the relationship. The multiplier $\:{K}_{0}$ controls the overall elevation of the curve and represents the baseline likelihood of low-intensity events, while the slope $\:K$ dictates how rapidly hazard decreases with increasing velocity. Steeper negative slopes imply rarer high-velocity floods, whereas flatter curves indicate comparatively more frequent extremes. Overall, the fitted parameters allow hazard behaviour to be quantified and compared across different conditions or scenarios.

### Flood vehicle vulnerability

**Fig. 5:**
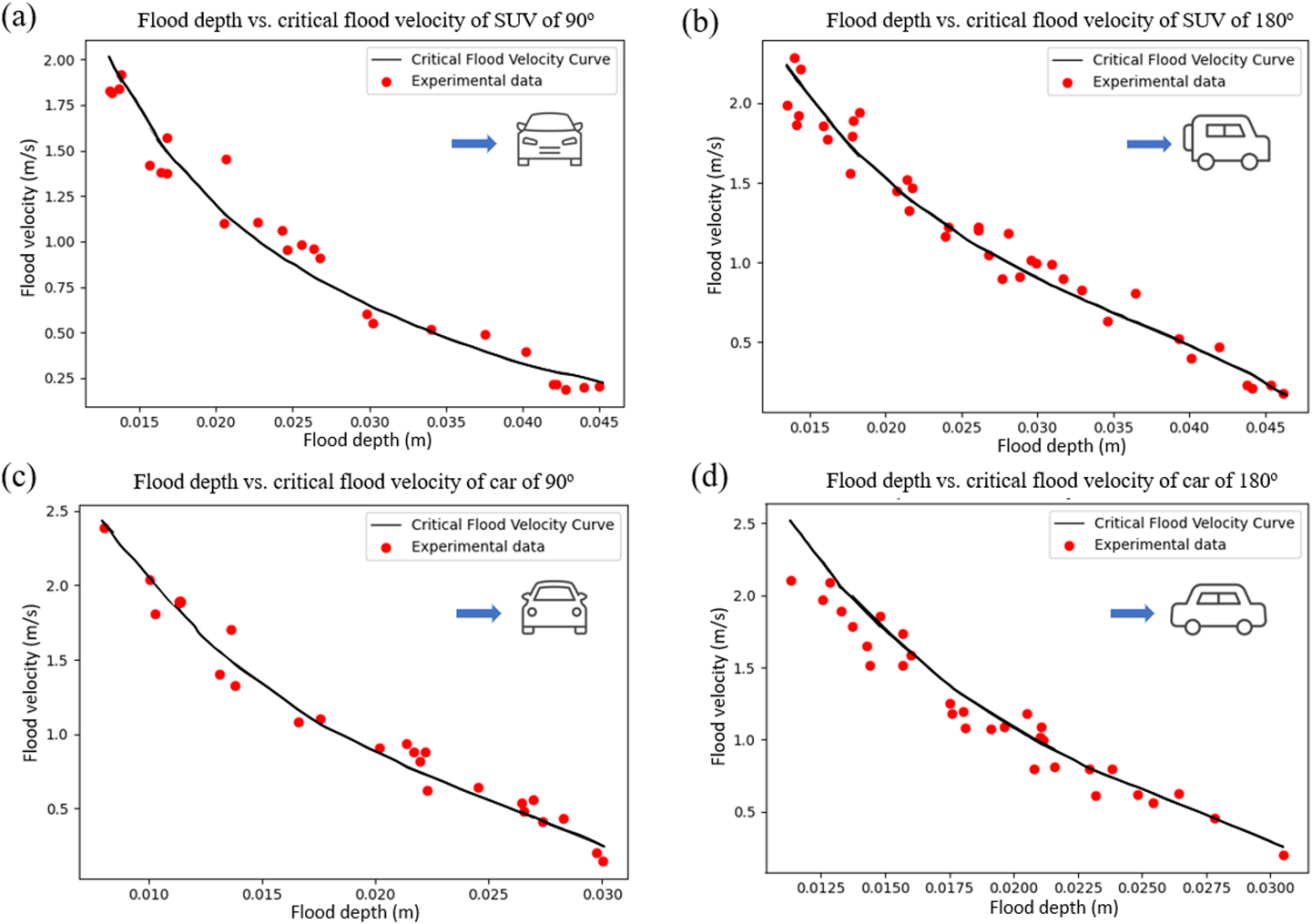
Critical flood velocity vs. depth for die-cast vehicle models (1:14) ; **a** perpendicular incoming flood for SUV stability; **b** parallel incoming flood for SUV stability; **c** perpendicular incoming flood for car stability; **d** parallel incoming flood for car stability (digitised and adapted from Xia et al. (2013)

Two types of vehicles are considered herein: (i) “SUVs”, which also represent emergency vehicles (e.g. ambulances); (ii) “cars”, which refers to regular private passenger vehicles (see Sect. 2.3). The calculation of fragility in Sect. 2.3 requires the logarithmic mean and logarithmic standard deviation of flood damage to vehicles. Equations 5 and 6 provide the central values. The standard deviation is obtained from the experimental residual. Figure 5 shows the digitisation of the experimental data from the literature (Xia et al. 2013) for the relationship between flood depth and critical flood velocity for two vehicle types, SUVs and cars, in water flow directions parallel and perpendicular to the vehicle. For the four combinations of vehicle type and incoming flood direction shown in Fig. 5 (SUV and car, with flow perpendicular and parallel to the vehicle), the fitted critical-velocity relationships reproduce the experimental data very closely, with coefficients of determination of $\:{R}_{\mathrm{SUV},{90}^{\circ\:}}^{2}\approx\:0.91$, $\:{R}_{\mathrm{SUV},0/{180}^{\circ\:}}^{2}\:\approx\:0.92$, $\:{R}_{\mathrm{car},{90}^{\circ\:}}^{2}\:\approx\:0.86$, and $\:{R}_{\mathrm{car},0/{180}^{\circ\:}}^{2}\:\approx\:0.81$, respectively, indicating an excellent agreement between the theoretical curves and the flume measurements. The fits for the SUV cases exhibit slightly higher coefficients of determination than those for the car, but all four $\:{R}^{2}$values are greater than 0.8, providing a high level of confidence in the fitted relationships. It is important to note that the vehicles used in the experiment were not full-sized real vehicles; instead, die-cast models at a scale of 1:14 were employed, as described by Xia et al. (2013). Although scaled-down vehicle models were used in the experiment, the residuals and standard deviation of the experimental results are not affected by the use of these smaller models. All geometric and hydraulic quantities were scaled proportionally in the vehicle stability experiment of Xia et al. (2013); therefore, even though a vehicle model was used, the simulated scenario can still reflect the stability of a real vehicle in a flood.

**Fig. 6:**
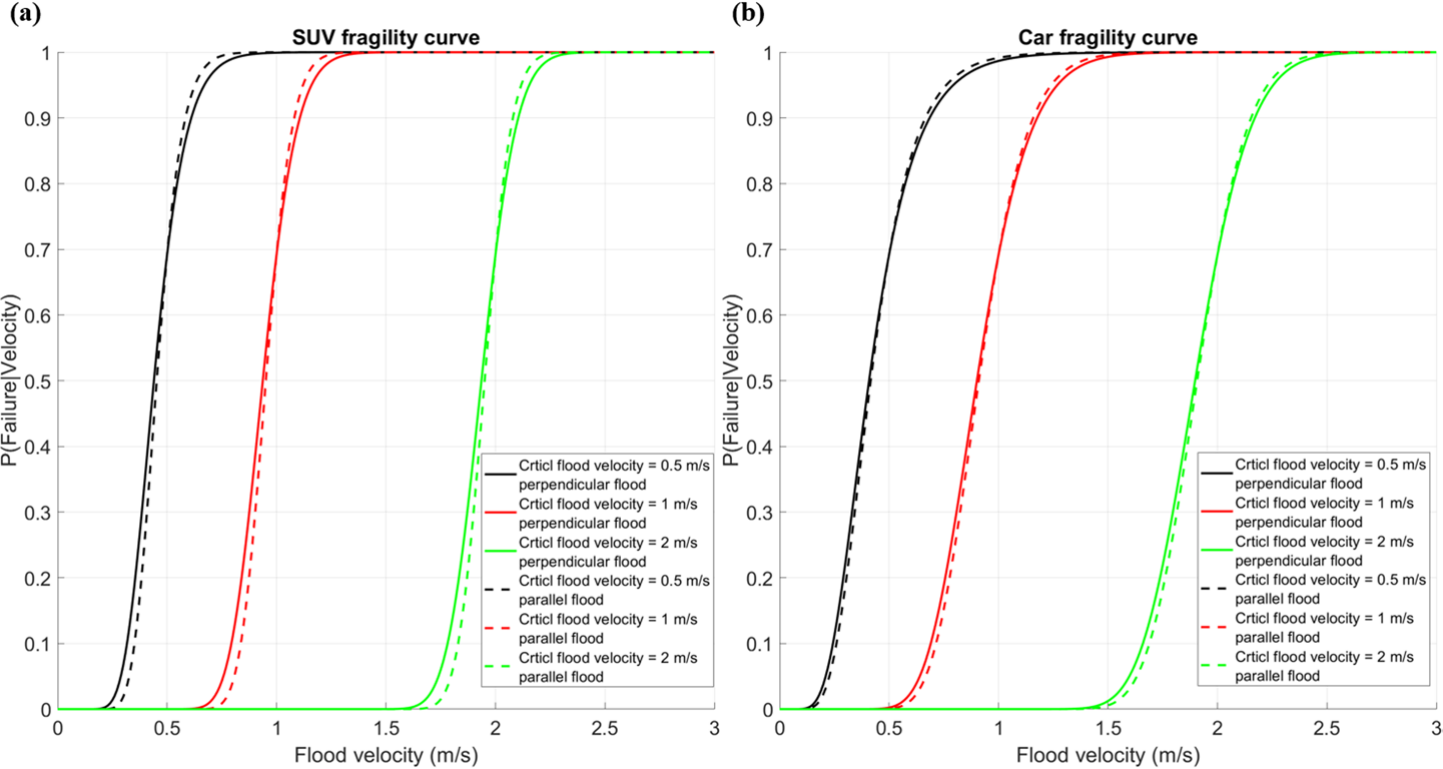
Fragility curves of vehicles at different critical flood velocities; **a** fragility curve of SUV under perpendicular (solid lines) and parallel (dash lines) incoming floods; **b** fragility curve of Car under perpendicular (solid lines) and parallel (dash lines) incoming flood

Table 2 lists the standard deviation for the different experiments. The results indicate that for both SUVs and cars, the standard deviation for parallel incoming flood is smaller than for perpendicular incoming flood. Figure 6 presents the fragility curves for SUVs and cars at different critical flood velocities. When the critical flood velocity is low, the fragility of SUVs and cars is essentially the same. Differences arise at higher flow velocities.

**Table 2:** Standard deviation of SUV and car under different incoming flood directions

Incoming flood direction	SUV	Car
perpendicular	0.13 m/s	0.16 m/s
parallel	0.11 m/s	0.15 m/s

### Flood risk maps

According to Eq. 1, flood hazard and fragility can be convoluted to obtain the flood risk for each return period. Finally, the flood risk for each return period is weighted to obtain the final annual flood risk, and the annual probability of failure of each link can be derived. Supplementary 1 and Supplementary 2 present the maps of risk at each flood return period for perpendicular incoming flood and parallel incoming flood for both SUVs and cars. As can be seen from both Supplementary 1 and Supplementary 2, as the flood return period increases, the probability of road functionality loss significantly rises for both SUVs and cars, with an increasing number of roads being affected by flooding. It is also worth noting that in any flood return period, the probability of failure of roads in the central area of the road network of Bristol, i.e., the area close to the River Avon and River Frome, is higher than that of other roads. In addition, comparing the failure probabilities of SUVs and cars shows that the flood risk of cars is significantly higher than that of SUVs under the same flood intensity. This result confirms that SUVs have a higher critical flood velocity than cars under the same flood depth (Martínez-Gomariz et al. 2017). Moreover, this result indicates that, at the same flood intensity, the road network maintains a higher level of transitability for emergency vehicles than for private passenger vehicles. In other words, emergency vehicles exhibit higher roadworthiness in a flood.

**Fig. 7:**
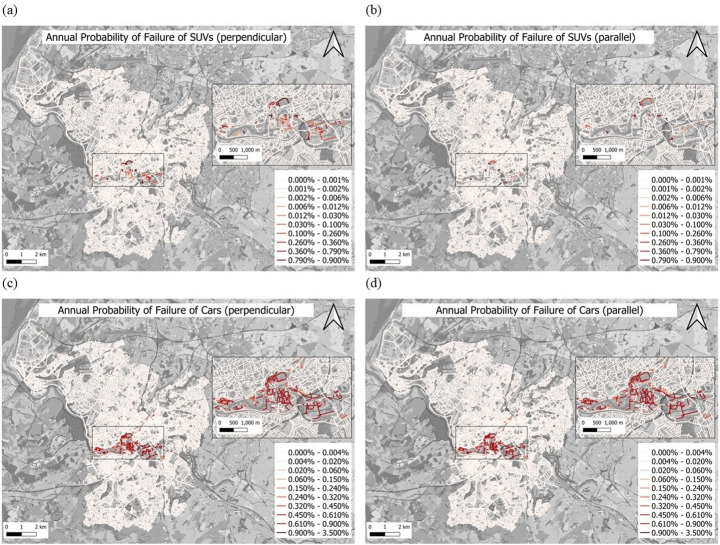
Flood risk maps; annual probability of failure for SUVs **a** under perpendicular and **b** under parallel incoming flood; annual probability of failure for cars **c** under perpendicular and **d** parallel incoming flood

Figure 7 shows the map of the annual probability of failure after weighting. The weighted annual flood risk map is independent of specific hazards and can capture the risk scenarios for potential flooding. Figure 7(a) and Fig. 7(b) are the annual probability of failure maps of the road network for SUVs of perpendicular and parallel incoming flood directions, and Fig. 7(c) and Fig. 7(d) are the annual probability of failure maps of the road network for cars of perpendicular and parallel incoming flood directions. Also, in this case, the roads in Bristol’s city centre are shown to be at a higher risk of flooding. This result confirms that the river is the primary source of flooding in Bristol. Additionally, comparing the weighted annual flood risk maps for SUVs and cars, the results clearly show that roads are more likely to lose functionality for cars during floods. From another perspective, the transitability of emergency vehicles during floods is consistently higher than that of private passenger vehicles.

The flood risk-weighted maps in Fig. 7 indicate a lower probability of failure for roads used by SUVs, demonstrating that SUVs have better flood roadworthiness in the flood. For SUVs, the perpendicular incoming flood presents a higher risk (Fig. 7a) than the parallel incoming flood (Fig. 7b). This result indicates that the stability damage caused to SUVs is greater when the floodwater approaches perpendicularly compared to parallel incoming floods. It also suggests that not only are common flood intensity measures (such as flood depth or velocity) fundamental for assessing road network flood risk, but the direction of flood velocity also dominates the outcomes of these assessments. For cars, the probability of failure maps show similar results regardless of whether the incoming flood direction is perpendicular or parallel (Fig. 7(c) and Fig. 7(d)). This result indicates that, for small vehicles such as cars, the flood direction does not significantly affect the failure probability; instead, flood intensity is the dominant factor. Additionally, it suggests that cars are less resistant to flooding and that roadworthiness is more restricted. It is important to note that the results in Fig. 7 represent the probability of functional loss for each road under the annual flood intensity for SUVs and cars. In other words, even though some roads may exhibit a high flood risk (red) in Fig. 7, it does not mean that these roads will lose functionality during an annual flood. It is just that high-flood-risk roads are more likely to exceed the limit state than low-flood-risk roads. Therefore, as explained above, the probability of functional road loss is lower during flood events, and the usability of emergency rescue vehicles is higher. However, this does not imply that emergency vehicles can always navigate flooded roads; instead, they demonstrate better roadworthiness than private passenger vehicles in flood scenarios.

### Transportation functionality assessment

**Fig. 8:**
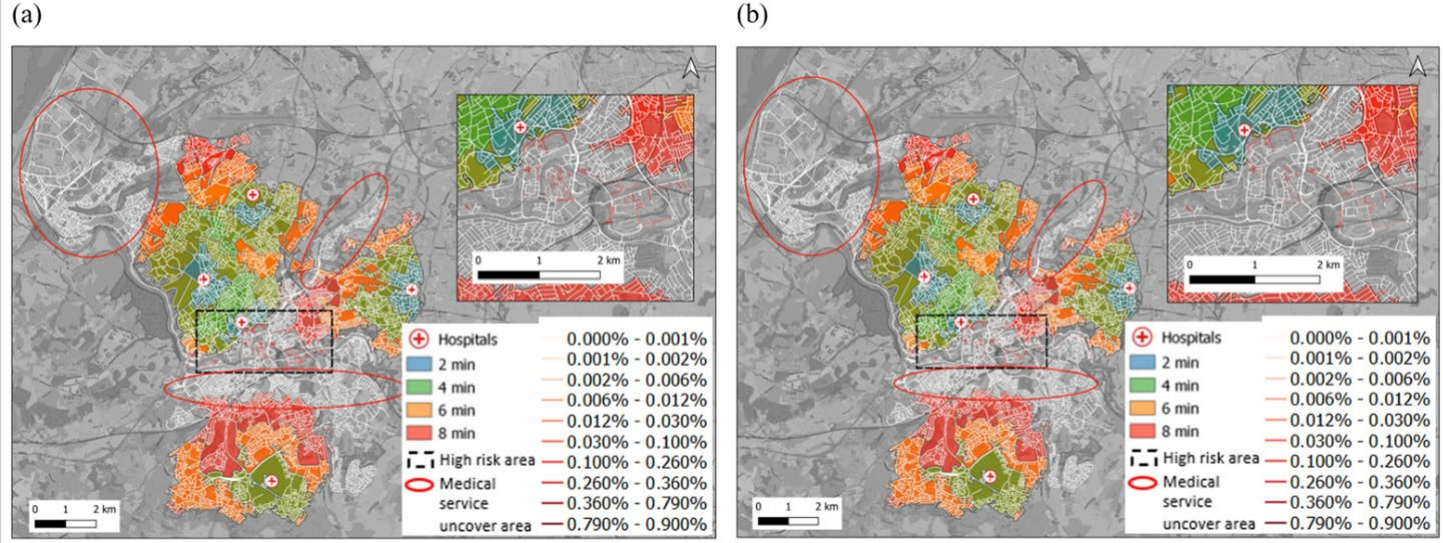
Isochrones of emergency vehicle travel times from the hospitals (shown in blue, green, orange, and red polygons) and the isochrone uncoverage areas (shown in red circles); **a** emergency vehicle reachable and unreachable areas when the road risk caused by perpendicular incoming flood; **b** emergency vehicle reachable and unreachable areas when the road risk caused by parallel incoming flood

This section investigates flood risk isochrones for hospital-based road networks, focusing on emergency medical service accessibility. After evaluating the flood risk and the annual probability of road failure under flooding conditions, it is necessary to incorporate road failures into traffic to assess the functional loss of the road network. A traffic indicator that can be used to evaluate the performance of road networks is the accessibility of vehicles at the regional level, which is represented by isochrones (see Sect. 2.4). In this study, all hospitals in Bristol with emergency ambulance services are considered as the starting points (origins) of vehicles in the isochrone. Travel time-based network performance is used to generate the road network’s isochrones. The deterioration of road network performance depends on the functional loss of each road. The functional loss of each road during a flood is mainly manifested as a reduction in its design travel speed. The decrease in road design speed is quantified by deriving the probability of failure from the flood risk map in Sect. 3.1. Although the probability of failure is a measure of the likelihood of a road losing its functional capacity, the percentage reduction in design speed of a road is calculated using the probability of failure of each specific link. Figure 8 shows the isochrone for all hospitals in Bristol for emergency services, accounting for reduced capacity. Since ambulances are typically large vehicles (comparable to SUVs), they are the only vehicle type considered here. According to the British new ambulance standard, ambulances are required to arrive at the patient’s location within 8 min of departure from the hospital (Coles et al. 2017; Green et al. 2017; CHANGE 2022). In this study, the 8-minute isochrone represents the maximum acceptable timeframe for ambulance response. To investigate the accessibility of the road network in more detail, the 8-minute isochrone is divided into four intervals of 2, 4, 6, and 8 min.

Figures 8(a) and (b) present the emergency medical service isochrones for all hospitals in Bristol under perpendicular and parallel incoming flood directions, respectively. Overall, the isochrones exhibit only minor differences between the two directions. This outcome may appear inconsistent with the results in Sect. 3.2, where SUVs showed greater instability under perpendicular flood flows. However, the spatial context clarifies the discrepancy: the Bristol city centre lies at a lower elevation than the surrounding areas, meaning that flooding affects the northern and southern road networks similarly regardless of flow direction. As a result, hospitals in these regions exhibit nearly identical accessibility patterns in both flood scenarios, resulting in the similar isochrones observed in Fig. 8. Even under these conditions, the maps reveal significant coverage limitations. In high-risk central areas, no hospital can reach all locations within 8 min. Furthermore, parts of northwest Bristol, the area south of the high-risk zone, and locations along the River Frome also fall outside the 8-minute isochrones. Although these latter areas are not highly flood-prone, their limited emergency accessibility during flooding suggests that ambulance mobility could still be compromised during future events.

## Discussion and innovation

Flood risk maps indicate the probability that a road network will reach a limit state. A higher limit-state probability implies greater vulnerability to flooding and a higher risk of functional loss. Deterministic approaches assess risk for each flood return period independently. In contrast, probabilistic approaches integrate risks across multiple return periods to produce a flood risk map that is not tied to a single scenario.

Probabilistic methods offer several advantages over deterministic ones. First, they do not rely on the selection of a specific return period; deterministic assessments, by contrast, require defining one or more scenarios (often a worst-case event). Second, probabilistic maps quantify the likelihood that roads will be affected by flooding, enabling the identification of vulnerable links even when flood events are uncertain. Deterministic outputs can only provide qualitative risk judgements for individual scenarios. Third, because the probabilistic method produces a single map summarising the probability of flood impact on roads, it delivers more comprehensive and actionable information for disaster management, supporting both risk reduction before flooding and efficient recovery afterwards.

The contributions of this study can be summarised in three key advances. First, vehicle fragility under flooding is quantified while explicitly incorporating model uncertainty derived from previous experimental studies. Here, fragility refers to the probability that a vehicle becomes unstable at a given level of flood intensity. Second, we integrate flood hazard with road fragility through a convolution process and then translate the resulting flood risk into traffic performance impacts, a relatively new direction in flood risk assessment. Previous research has primarily focused on structural damage and associated economic or human losses (e.g., De Risi et al. 2013), rather than on transport-system functionality. Third, we adopt a hazard-agnostic approach, which can be generalised to other types of disruptions affecting physical network components (Beevers et al. 2022). This approach increases the flexibility and robustness of the analysis and allows the same methodology to be used for assessing network improvements or alternative scenario conditions.

This work also offers practical value. Time-based isochrones provide a clear representation of traffic vulnerability during floods and can support emergency planning, response, and recovery. For instance, the isochrone analysis suggests that emergency vehicle locations could be optimised to improve coverage during flood events in Bristol. In this way, isochrones can enhance transport network resilience.

However, several limitations must be acknowledged. Isochrones represent static accessibility and do not account for interactions between vehicles or changes in traffic flow over time. The analysis also assumes that vehicles travel at the maximum permitted speed, neglecting delays from traffic signals and congestion. Consequently, the resulting isochrone extents should be interpreted as optimistic, upper-bound estimates, though the assumption remains reasonable for emergency vehicles, which can proceed without signal restrictions, as supported by existing literature (e.g., Pregnolato et al. 2016). Finally, the study utilises only publicly available information and does not incorporate specific contingency measures that could be implemented when flooding is forecast.

## Conclusion

This study presents a probabilistic approach for evaluating flood risk maps of road networks, combining flood hazard maps for different return periods and flood fragility functions of vehicles. The flood risk of road networks is measured as the impact on traffic. The work focuses mainly on emergency medical vehicles. For the first time, this paper proposed vehicle fragility curves based on previous experimental results. Moreover, it proposed a methodology to ensure the fragilities are used in a hazard-agnostic fashion.

The flood risk is assessed for the study of Bristol. The main findings of this study are as follows. First, the flood risk of roads near rivers is high, as expected. Second, cars show a much higher flood risk compared to SUVs. Third, the isochrones computed for all the hospitals in the city offering emergency rescue functions show that ambulances may take more than 8 min to reach areas with high flood risk, deemed the ideal time for emergency services to arrive in case of injuries.

This study has a few limitations. A static approach was employed regarding the traffic simulation within the road network, focusing solely on determining origin-destination pairs to reflect accessibility within specific travel times. Furthermore, the study does not provide methods for modelling changes in traffic flow on each road or the mutual interactions among vehicles. Therefore, in future research, agent-based approaches should be employed to simulate changes in traffic flow on roads within flood risk scenarios. Additionally, by incorporating variations in the traffic flow within the road network, future studies could focus on the impact of intervention measures to enhance the flood resilience of road.

## Supplementary Information

Below is the link to the electronic supplementary material.

Supplementary Material 1

Supplementary Material 2

## References

[CR1] Akinsowon V (2021), September 24 Isochrone Map Generator: How to Create Isochrone Maps (& Why You Should) [Review of *Isochrone Map Generator: How to Create Isochrone Maps (& Why You Should)*]. *TravelTime*. https://traveltime.com/blog/free-isochrone-map-generator

[CR4] Al-Qadami EHH, Mustaffa Z, Shah SMH, Matínez-Gomariz E, Yusof KW (2021) Full-scale experimental investigations on the response of a flooded passenger vehicle under subcritical conditions. Nat Hazards. 10.1007/s11069-021-04949-6

[CR2] Alabbad Y, Mount J, Campbell AM, Demir I (2021) Assessment of transportation system disruption and accessibility to critical amenities during flooding: Iowa case study. Sci Total Environ 793:148476. 10.1016/j.scitotenv.2021.148476 34174595 10.1016/j.scitotenv.2021.148476

[CR3] Alexandros Efentakis, Grivas N, Lamprianidis G, Dieter Pfoser (2013) Georg Magenschab, &. Isochrones, traffic and DEMOgraphics. *Zenodo (CERN European Organization for Nuclear Research)*. 10.1145/2525314.2525325

[CR5] Apel H, Aronica GT, Kreibich H, Thieken AH (2008) Flood risk analyses—how detailed do we need to be? Nat Hazards 49(1):79–98. 10.1007/s11069-008-9277-8

[CR53] U.S. Army Corps of Engineers (2019) HEC-RAS River Analysis System: Hydraulic Reference Manual (5.0.7). Washington, DC

[CR6] ARUP (2020) *Bristol Avon Flood Strategy*. tech. Bristol City Council. Available at: https://democracy.bristol.gov.uk/documents/s57930/Appendix%20A%20Strategic%20Outline%20Case.pdf

[CR7] Bainbridge, L. (2021, November 13). What is an Isochrone Map? A Definition &; Examples.*TravelTime*. October 31, 2023, https://traveltime.com/blog/what-is-an-isochrone

[CR8] Bates PD, De Roo APJ (2000) A simple raster-based model for flood inundation simulation. J Hydrol 236(1–2):54–77. 10.1016/s0022-1694(00)00278-x

[CR9] Bates PD, Savage J, Wing O, Quinn N, Sampson C, Neal J, Smith A (2023) A climate-conditioned catastrophe risk model for UK flooding. Nat Hazards Earth Syst Sci 23(2):891–908. 10.5194/nhess-23-891-2023

[CR10] Beevers L, McClymont K, Bedinger M (2022) A hazard-agnostic model for unpacking systemic impacts in urban systems. Civil Eng Environ Syst 39(3):224–241. 10.1080/10286608.2022.2083112

[CR11] Chang L, Kang J, Su M (2009) Depth-Damage Curve for Flood Damage Assessments Industrial and Commercial Sectors. *In: Proceedings of the 4th IASME/WSEAS international conference on water resources, hydraulics & hydrology*

[CR12] CHANGE (2022) The New Ambulance Standards [PDF] (pp. 1–5). Retrieved 25 March 2024, from https://www.england.nhs.uk/wp-content/uploads/2017/07/new-ambulance-standards-easy-read.pdf

[CR13] Coles D, Yu D, Wilby RL, Green D, Herring Z (2017) Beyond ‘flood hotspots’: modelling emergency service accessibility during flooding in York, UK. J Hydrol 546:419–436. 10.1016/j.jhydrol.2016.12.013

[CR14] De Risi R (2023), January *Structural Reliability & Risk-Based design Part 2: Uncertainties, why bother? Basics of Probability & Statistics*. Lecture

[CR16] De Risi R, Jalayer F, De Paola F, Iervolino I, Giugni M, Topa ME, Mbuya E, Kyessi A, Manfredi G, Gasparini P (2013) Flood risk assessment for informal settlements. Nat Hazards 69(1):1003–1032. 10.1007/s11069-013-0749-0

[CR17] De Risi R, Jalayer F, De Paola F (2015) Meso-scale hazard zoning of potentially flood-prone areas. J Hydrol 527:316–325. 10.1016/j.jhydrol.2015.04.070

[CR18] De Risi R, Jalayer F, De Paola F, Lindley S (2018a) Delineation of flooding risk hotspots based on digital elevation model, calculated and historical flooding extents: the case of Ouagadougou. Stoch Env Res Risk Assess 32(6):1545–1559. 10.1007/s00477-017-1450-8

[CR15] De Risi R, De Paola F, Turpie J, Kroeger T (2018b) Life cycle cost and return on investment as complementary decision variables for urban flood risk management in developing countries. Int J Disaster Risk Reduct 28:88–106

[CR19] De Risi R, Jalayer F, De Paola F, Carozza S, Yonas N, Giugni M, Gasparini P (2020) From flood risk mapping toward reducing vulnerability: the case of addis Ababa. Nat Hazards 100(1):387–415. 10.1007/s11069-019-03817-8

[CR20] Dias P, Arambepola NMSI, Weerasinghe K, Weerasinghe KDN, Wagenaar D, Bouwer LM, Gehrels H (2018) Development of damage functions for flood risk assessment in the City of Colombo (Sri Lanka). Procedia Eng 212:332–339. 10.1016/j.proeng.2018.01.043

[CR21] Dong B, Xia J, Li Q, Zhou M (2022a) Risk assessment for people and vehicles in an extreme urban flood: case study of the 7.20 flood event in Zhengzhou, China. Int J Disaster Risk Reduct 80:103205. 10.1016/j.ijdrr.2022.103205

[CR22] Dong S, Gao X, Mostafavi A, Gao J (2022b) Modest flooding can trigger catastrophic road network collapse due to compound failure. Commun Earth Environ 3(1). 10.1038/s43247-022-00366-0

[CR23] Evans B, Lam A, West C, Ahmadian R, Slobodan Djordjević, Chen A, Pregnolato M (2024) A combined stability function to quantify flood risks to pedestrians and vehicle occupants. Sci Total Environ 908:168237–168237. 10.1016/j.scitotenv.2023.168237 37926250 10.1016/j.scitotenv.2023.168237

[CR24] Galasso C, Pregnolato M, Parisi F (2021) A model taxonomy for flood fragility and vulnerability assessment of buildings. Int J Disaster Risk Reduct 53:101985

[CR25] Green D, Yu D, Pattison I, Wilby R, Bosher L, Patel R, Thompson P, Trowell K, Draycon J, Halse M, Yang L, Ryley T (2017) City-scale accessibility of emergency responders operating during flood events. Nat Hazards Earth Syst Sci 17(1):1–16. 10.5194/nhess-17-1-2017

[CR26] He K, Risi RD, Pregnolato M, Carhart N, Neal J (2023) Graph-based Framework for Road Network Performance and Flood Risk Assessment (pp. 1–7). *In 14th International Conference on Applications of Statistics and Probability in Civil Engineering*. Dublin; ICASP14

[CR27] He K, Risi RD, Pregnolato M, Carhart N, Neal J (2024) Functionality assessment of road network combining flood roadworthiness and graph topology. Transportation research part D: transport and environment. Manuscript submitted for publication

[CR28] Hirai S, Yasuda T (2018) Risk assessment of aggregate loss by storm surge inundation in Ise and Mikawa bay. Coastal Eng Proc 1(36):35. 10.9753/icce.v36.risk.35

[CR29] Jalayer F, De Risi R, De Paola F, Giugni M, Manfredi G, Gasparini P, Topa ME, Yonas N, Yeshitela K, Nebebe A, Cavan G, Lindley S, Printz A, Renner F (2014) Probabilistic GIS-based method for delineation of urban flooding risk hotspots. Nat Hazards 73:975–1001. 10.1007/s11069-014-1119-2

[CR30] Jalayer F, Carozza S, De Risi R, Manfredi G, Mbuya E (2016) Performance-based flood safety-checking for non-engineered masonry structures. Eng Struct 106:109–123

[CR32] Kalantari Z, Cavalli M, Cantone C, Crema S, Destouni G (2017) Flood probability quantification for road infrastructure: Data-driven spatial-statistical approach and case study applications. Sci Total Environ 581–582:386–398. 10.1016/j.scitotenv.2016.12.147 10.1016/j.scitotenv.2016.12.147 28062101

[CR31] Karambiri H, Tazen F, Traore MB, Mounirou LA, Coulibaly G, Traore K (2018) *Build relationship between flood depth and likely damage (depth-damage curves)*. AMMA-2050

[CR33] Kasmalkar IG, Serafin KA, Miao Y, Bick IA, Ortolano L, Ouyang D, Suckale J (2020) When floods hit the road: resilience to flood-related traffic disruption in the San Francisco Bay area and beyond. Sci Adv 6(32). 10.1126/sciadv.aba2423 10.1126/sciadv.aba2423 PMC7406370 32821823

[CR34] Kinyua GJ (2018) *Flood Risk Analysis Using the Risk Curves in Mathare Valley Nairobi Kenya*. https://www.researchgate.net/publication/326734183_FLOOD_RISK_ANALYSES_USING_THE_RISK_CURVES_IN_MATHARE_VALLEY_NAIROBI_KENYA

[CR35] Kreibich H, Piroth K, Seifert I, Maiwald H, Kunert U, Schwarz J, Merz B, Thieken AH (2009) Is flow velocity a significant parameter in flood damage modelling? Nat Hazards Earth Syst Sci 9(5):1679–1692

[CR36] Lazzarin T, Viero DP, Molinari D, Ballio F, Defina A (2022) Flood damage functions based on a single physics- and data-based impact parameter that jointly accounts for water depth and velocity. J Hydrol 607:127485. 10.1016/j.jhydrol.2022.127485

[CR37] Li T, Lee G, Kim G (2021) Case study of urban flood Inundation—Impact of Temporal variability in rainfall events. Water 13(23):3438. 10.3390/w13233438

[CR38] Liu T, Wang Y, Yu H, Chen Y (2022) Using statistical functions and hydro-hydraulic models to develop human vulnerability curves for flash floods: the flash flood of the Taitou catchment (China) in 2016. Int J Disaster Risk Reduct 73:102876. 10.1016/j.ijdrr.2022.102876

[CR39] Martínez-Gomariz E, Gómez M, Russo B, Djordjević S (2017) A new experiments-based methodology to define the stability threshold for any vehicle exposed to flooding. Urban Water J 14(9):930–939. 10.1080/1573062x.2017.1301501

[CR40] McKenzie H (2022) How to create and use Isolines with CARTO. *CART*. Available at: https://carto.com/blog/creating-isolines-with-carto [Accessed 24 Mar. 2024]

[CR41] Michael Havbro Faber (2012) Statistics and probability theory. Springer Science & Business Media

[CR42] Papilloud T, Keiler M (2021) Vulnerability patterns of road network to extreme floods based on accessibility measures. Transp Res Part D: Transp Environ 100:103045. 10.1016/j.trd.2021.103045

[CR44] Pregnolato M, Ford A, Robson C, Glenis V, Barr S, Dawson R (2016) Assessing urban strategies for reducing the impacts of extreme weather on infrastructure networks. Royal Soc Open Sci 3(5):160023 10.1098/rsos.160023 PMC4892443 27293781

[CR43] Pregnolato M, Ford A, Glenis V, Wilkinson S, Dawson R (2017) Impact of climate change on disruption to urban transport networks from pluvial flooding. J Infrastruct Syst 23(4):04017015

[CR45] Pregnolato M, Winter AO, Mascarenas D, Sen AD, Bates P, Motley MR (2022) Assessing flooding impact to riverine bridges: an integrated analysis. Nat Hazards Earth Syst Sci 22(5):1559–1576

[CR46] Pyatkova K, Chen AS, Butler D, Vojinović Z, Djordjević S (2019) Assessing the knock-on effects of flooding on road transportation. J Environ Manage 244:48–60. 10.1016/j.jenvman.2019.05.013 31108310 10.1016/j.jenvman.2019.05.013

[CR47] Rebally A, Valeo C, He J, Saidi S (2021) Flood impact assessments on transportation networks: A review of methods and associated Temporal and Spatial scales. Front Sustainable Cities. 310.3389/frsc.2021.732181

[CR48] Seenath A (2018) Effects of DEM resolution on modeling coastal flood vulnerability. Mar Geodesy 41(6):581–604. 10.1080/01490419.2018.1504838

[CR49] Shu C, Xia J, Falconer RA, Lin B (2011) Incipient velocity for partially submerged vehicles in floodwaters. J Hydraul Res 49(6):709–717. 10.1080/00221686.2011.616318

[CR50] Śleszyński P, Olszewski P, Dybicz T, Goch K, Niedzielski MA (2023) The ideal isochrone: assessing the efficiency of transport systems. Res Transp Bus Manage 46:100779. 10.1016/j.rtbm.2021.100779

[CR51] Teo FY, Xia J, Falconer RA, Lin B (2012) Experimental studies on the interaction between vehicles and floodplain flows. Int J River Basin Manage 10(2):149–160. 10.1080/15715124.2012.674040

[CR52] Toda K, Ishigaki T, Ozaki T (2013) Experiments study on floating car in flooding. In: International Conference on Flood Resilience 2013 (ICFR 2013), Experiences in Asia and Europe, Exeter (UK)

[CR54] Victor Miguel Ponce, &, Simons DB (1977) Shallow wave propagation in open channel flow. J Hydraulics Div 103(12):1461–1476. 10.1061/jyceaj.0004892

[CR55] Wang N, Hou J, Du Y, Jing H, Wang T, Xia J, Gong J, Huang M (2021) A dynamic, convenient and accurate method for assessing the flood risk of people and vehicle. Sci Total Environ 797:149036. 10.1016/j.scitotenv.2021.149036 34311368 10.1016/j.scitotenv.2021.149036

[CR56] Wu X, Wang Z, Guo S, Lai C, Chen X (2018) A simplified approach for flood modeling in urban environments. Hydrol Res 49(6):1804–1816. 10.2166/nh.2018.149

[CR58] Xia J, Teo FY, Lin B, Falconer RA (2011) Formula of incipient velocity for flooded vehicles. Nat Hazards 58(1):1–14. 10.1007/s11069-010-9639-x

[CR57] Xia J, Falconer RA, Xiao X, Wang Y (2013) Criterion of vehicle stability in floodwaters based on theoretical and experimental studies. Nat Hazards 70(2):1619–1630. 10.1007/s11069-013-0889-2

[CR59] Xia J, Teo FY, Falconer RA, Chen Q, Deng S (2016) Hydrodynamic experiments on the impacts of vehicle blockages at bridges. J Flood Risk Manag 11. 10.1111/jfr3.12228

[CR60] Yang Q, Ma Z, Zhang S (2022) Urban pluvial flood modeling by coupling Raster-Based Two-Dimensional hydrodynamic model and SWMM. Water 14(11):1760. 10.3390/w14111760

[CR61] Zeng W, Fu C-W, Arisona SM, Erath A, Qu H (2014) Visualising mobility of public transportation system. IEEE Trans Vis Comput Graph 20(12):1833–1842. 10.1109/TVCG.2014.2346893 26356897 10.1109/TVCG.2014.2346893

[CR62] Zhang N, Alipour A (2019) Integrated framework for risk and resilience assessment of the road network under inland flooding. Transp Res Record: J Transp Res Board 2673(12):182–190. 10.1177/0361198119855975

