# Supplementary material for: Probabilistic functionality assessment of road networks for medical emergency vehicles during flooding
Source: Nat Hazards (Dordr). 2026 Feb 26;122(6):233. doi: 10.1007/s11069-026-08005-z (PMC12946296; doi:10.1007/s11069-026-08005-z)

**Probabilistic hazard-agnostic functionality assessment of transportation networks for medical emergency SUV during flooding (Supplementary Materials)**

Probability of failure maps of SUVs under both incoming flood directions are perpendicular and parallel


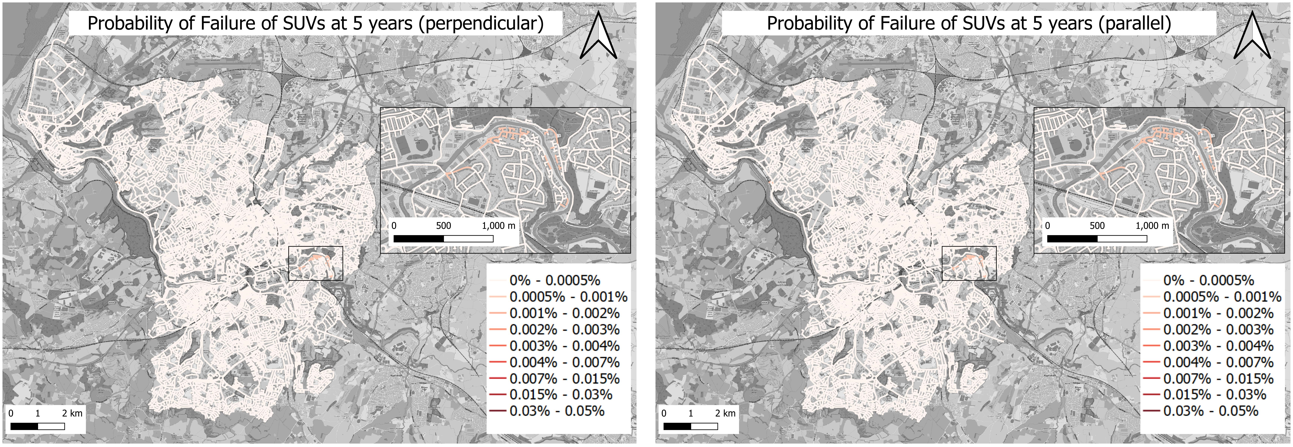


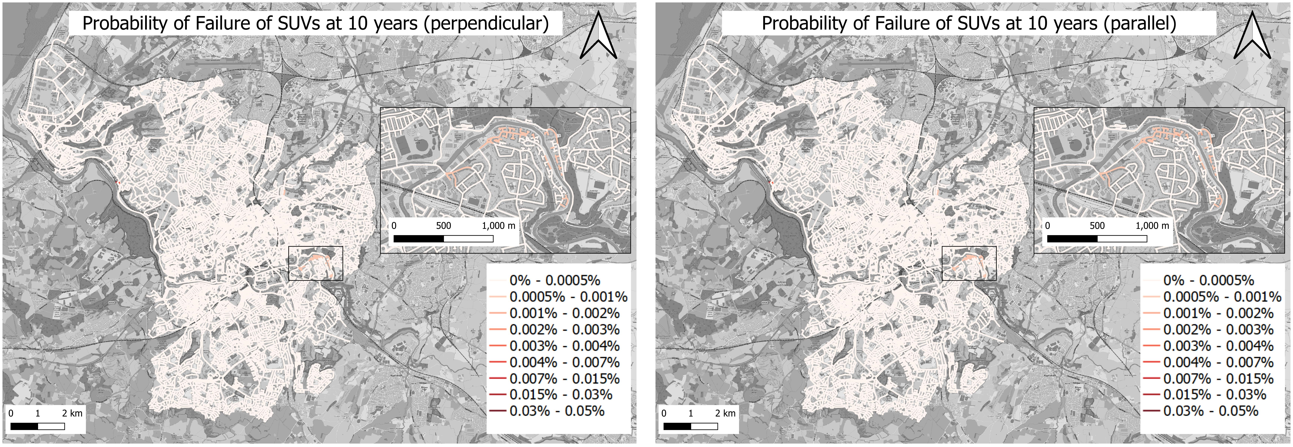


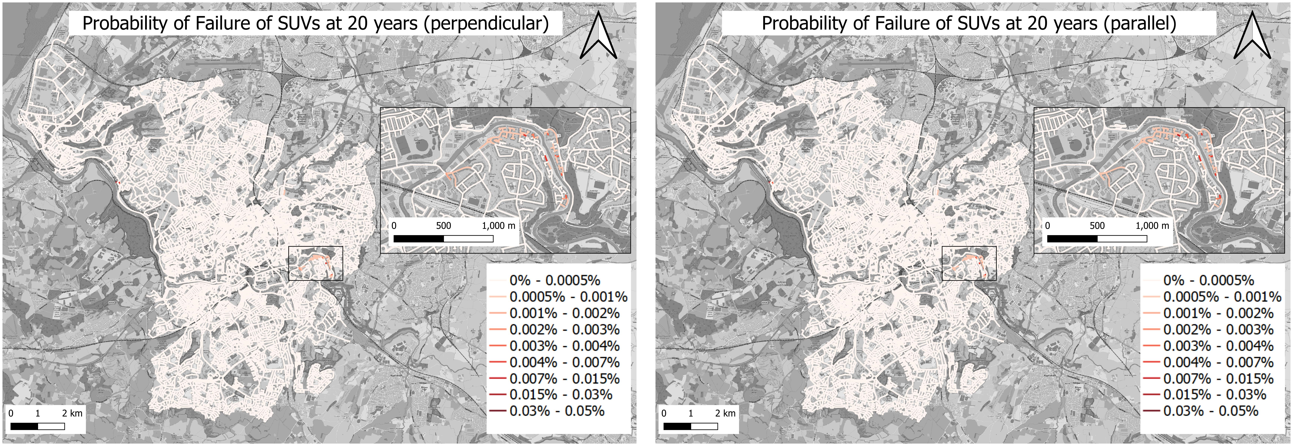


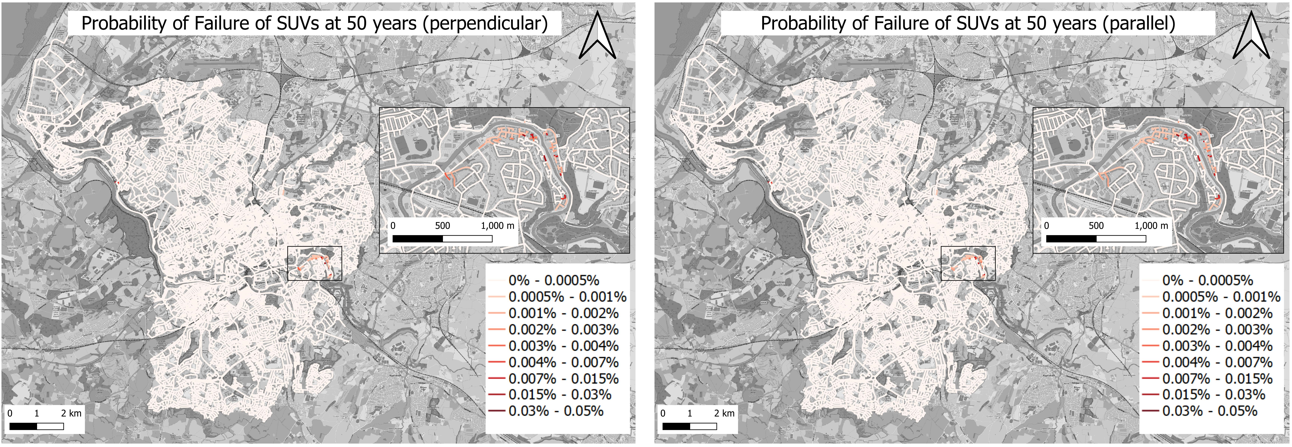


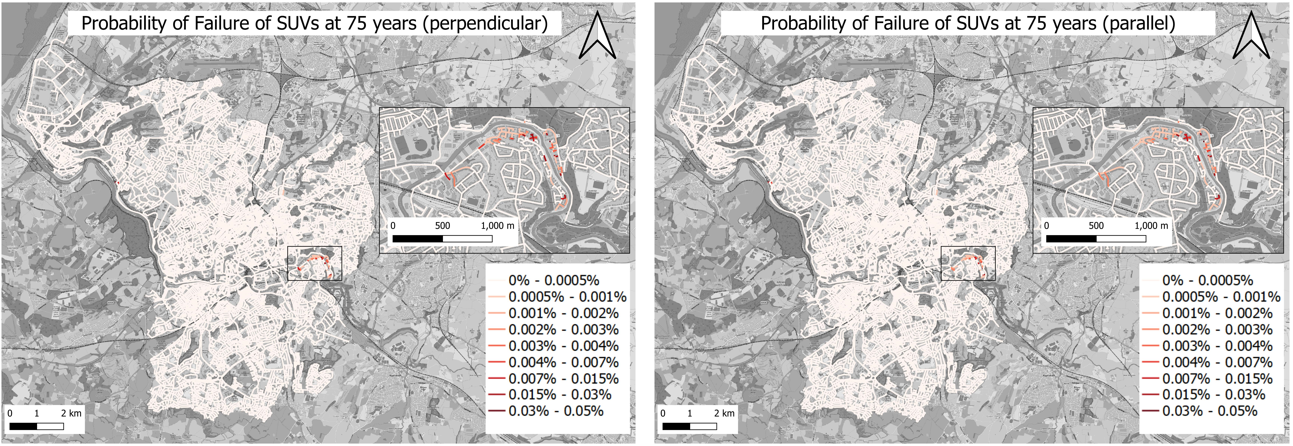


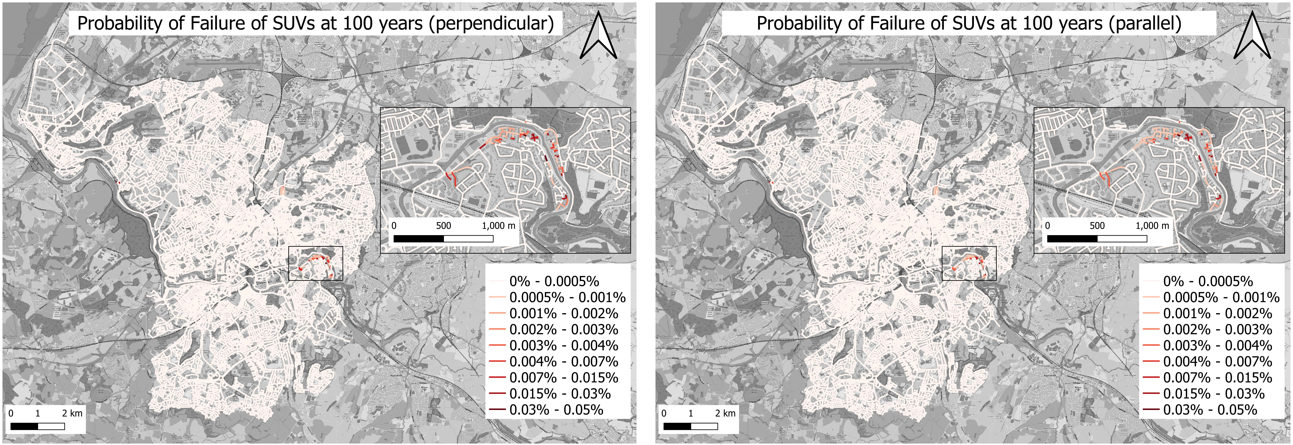


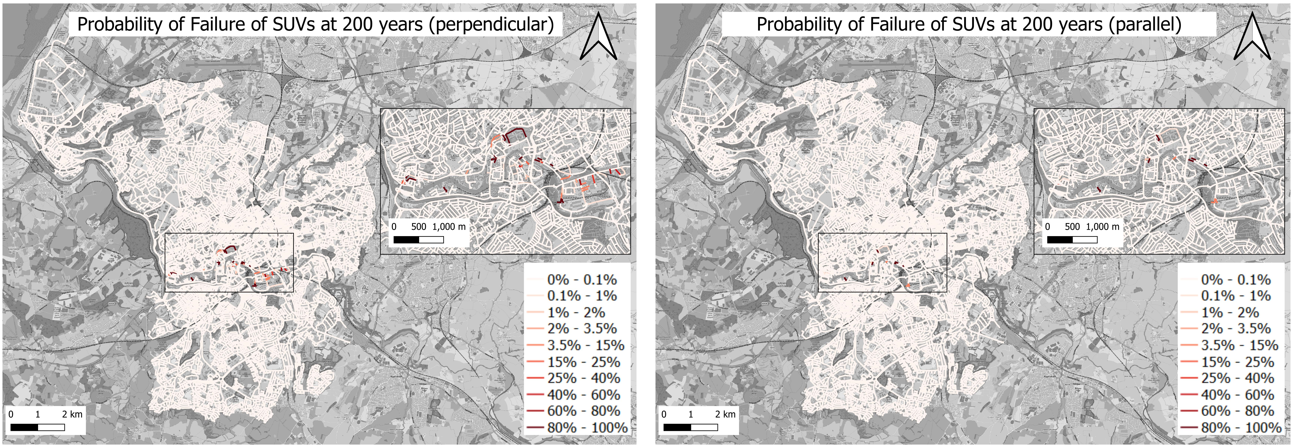


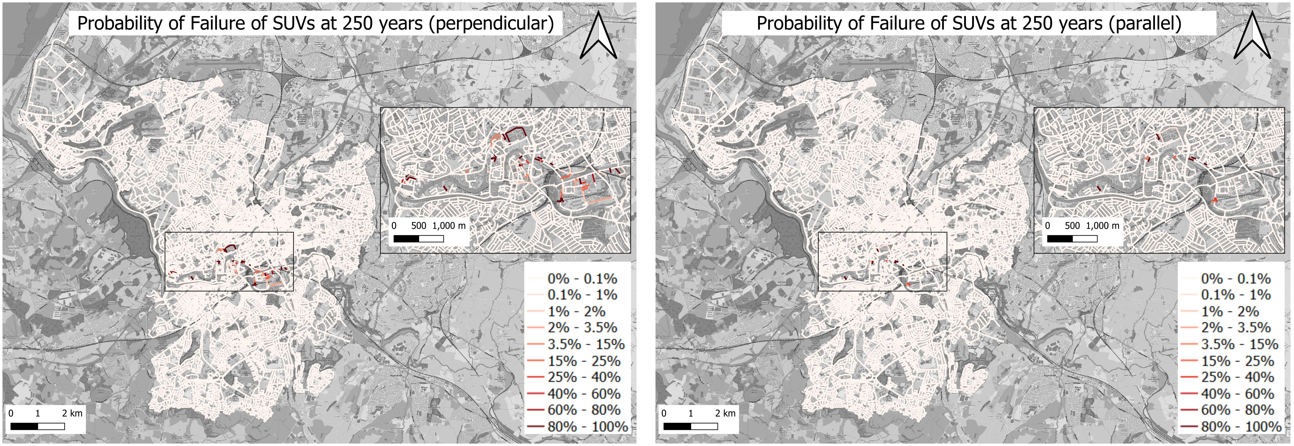


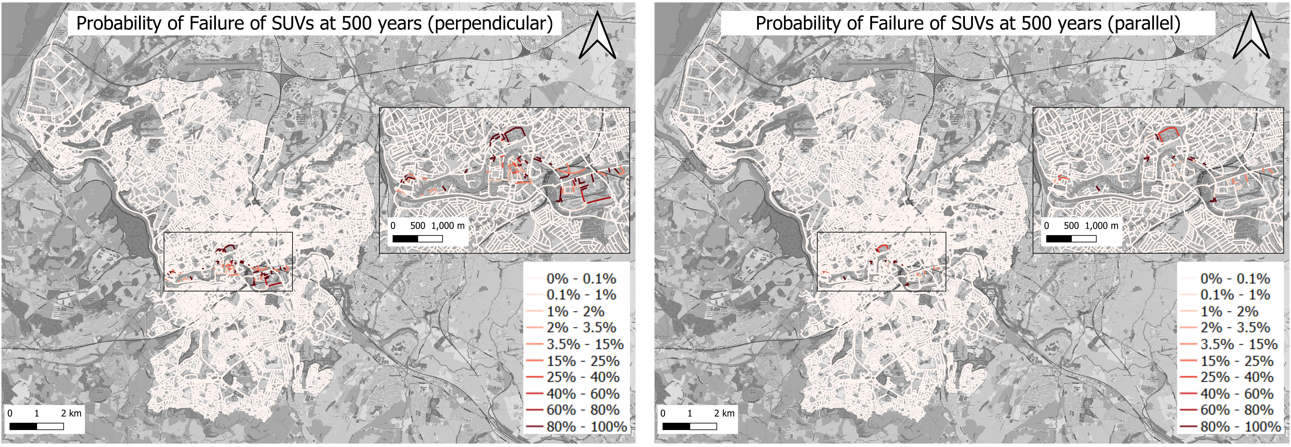


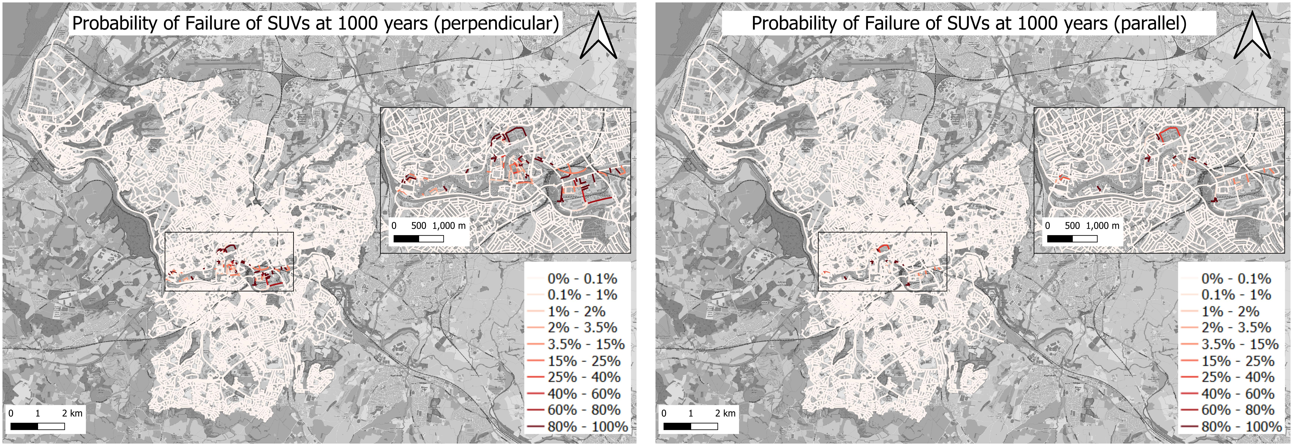

Supplement: Supplementary file 1 — Supplementary Material 1 [file 11069_2026_8005_MOESM1_ESM.docx]
